# 338. Multicenter Evaluation of Superinfection Occurrence and Impact on Clinical Outcomes in Patients with COVID-19

**DOI:** 10.1093/ofid/ofab466.539

**Published:** 2021-12-04

**Authors:** Taryn A Eubank, Katherine Perez, William L Musick, Kevin W Garey

**Affiliations:** 1 University of Houston, Houston, Texas; 2 Houston Methodist Hospital, Houston, Texas; 3 University of Houston College of Pharmacy, Houston, Texas

## Abstract

**Background:**

The coronavirus disease 2019 (COVID-19), caused by the novel severe acute respiratory syndrome coronavirus 2 (SARS-CoV-2), spread globally throughout late 2019. During this pandemic, concern for bacterial and fungal superinfections has been present during the treatment of these patients.

**Methods:**

Hospitalized, adult patients with laboratory confirmed and symptomatic COVID-19 disease admitted between March 12, 2020 and May 31, 2020 were eligible for inclusion in this study. Data was obtained from electronic medical records and the hospital system’s clinical surveillance program including demographics, comorbidities, hospitalization dates, laboratory values, mechanical ventilation, positive blood and respiratory cultures, treatment administration for COVID-19 as defined by the system’s fluid treatment algorithm, and discharge disposition. Outcomes of this analysis include overall bacterial and fungal superinfection occurrence rate within 28 days of admission, patient characteristics that correlate with a higher risk of a superinfection, and the effect on 28-day mortality.

Patient Population

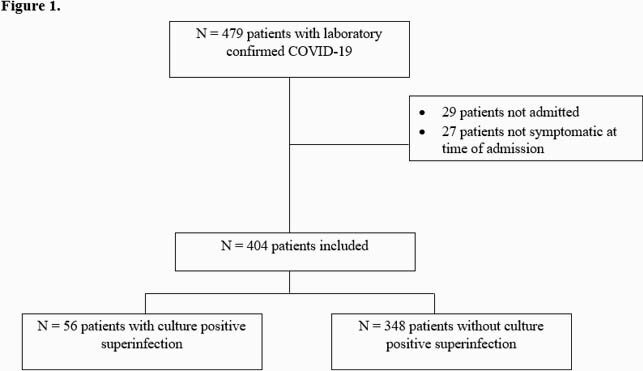

Flow diagram of patient inclusion.

**Results:**

A total of 404 patients were included in the study analyses of which 56 (13.9%) had a documented superinfection within 28-days from admission. The most common superinfection organisms observed were *Staphylococcus* spp. (36.9%), *Candida* spp. (16.7%), and *Klebsiella* spp. (13.1%). Mortality was significantly higher in patients with superinfections (12.1% vs 5.8%, *p* < 0.001). To best assess characteristics that place patients at a higher risk of superinfection, a backwards, stepwise, multivariable logistic regression was performed. Black ethnicity, chronic kidney disease, intensive care unit (ICU) upon admission, lymphocytopenia, and receipt of tocilizumab were found to more likely have a superinfection within 28-days from admission.

Baseline Characteristics

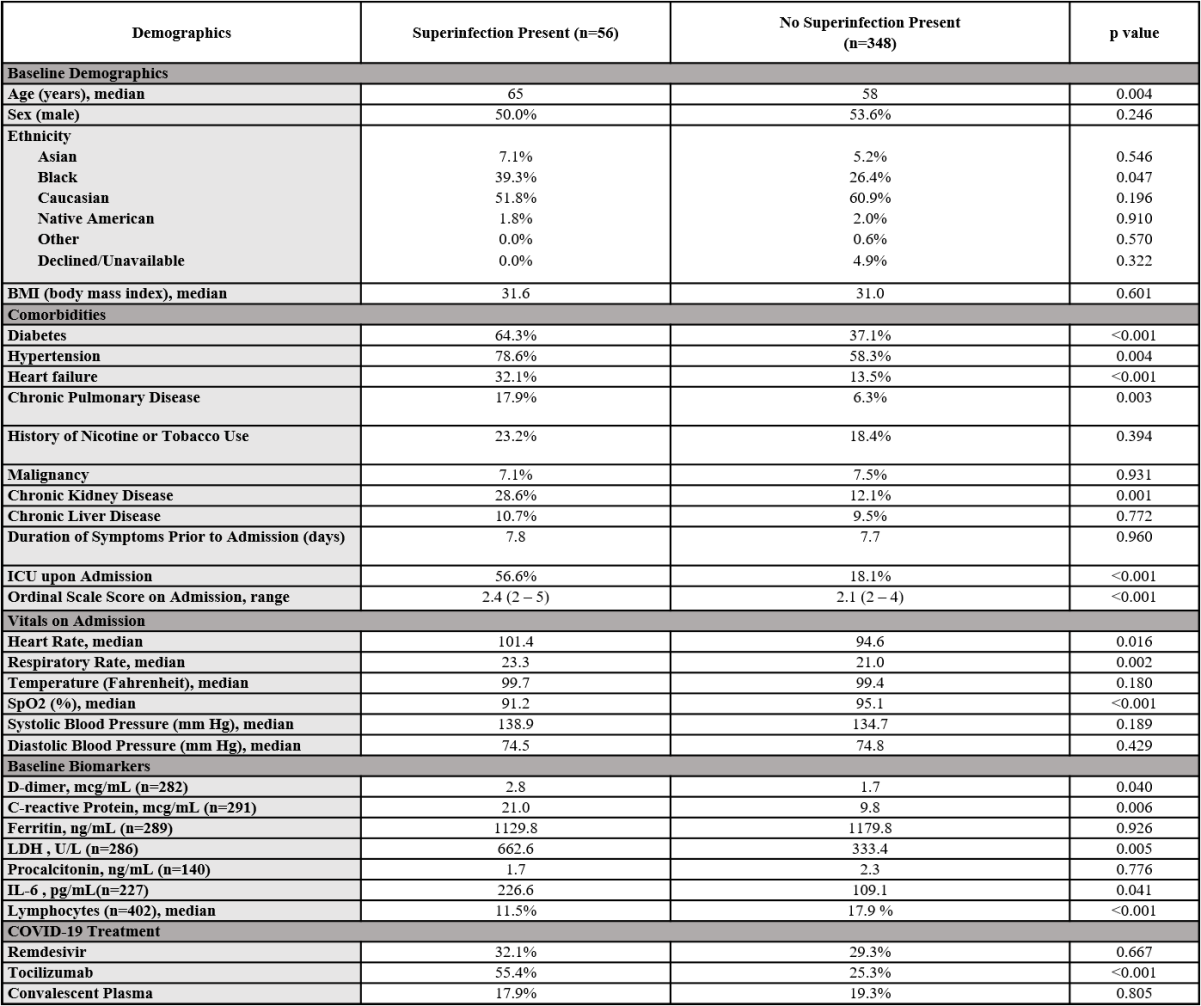

Comparison and analysis of baseline characteristics in patients with or without superinfection present.

28-day Mortality

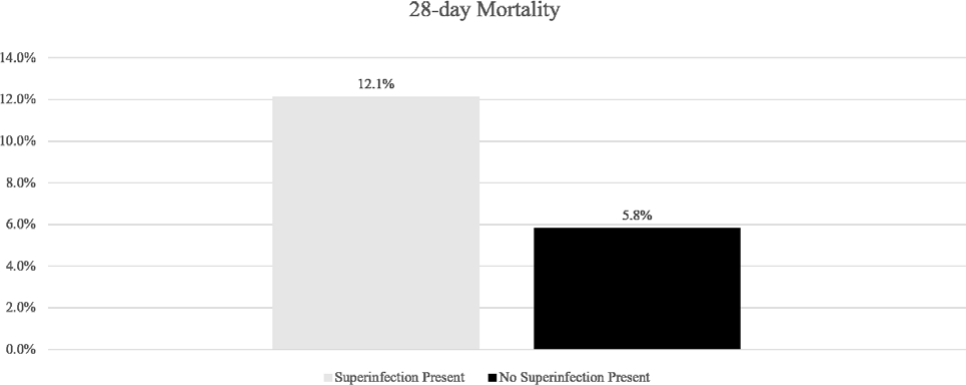

Day-28 mortality comparison in patients with or without superinfection. Mortality was observed in 7/58 patients with a superinfection versus 20/346 patients without superinfection present (p < 0.001).

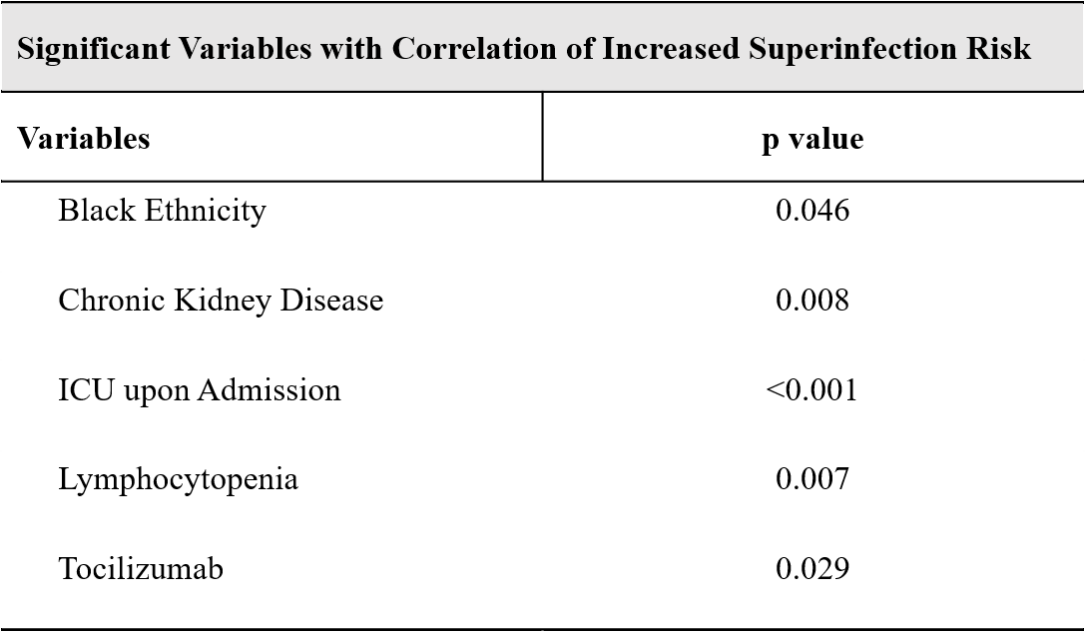

Multivariable analysis results for increased superinfection risk. All baseline characteristics with univariate analysis resulting in a p value of < 0.2 were included in the backwards, stepwise logistic regression model.

**Conclusion:**

In conclusion, our retrospective cohort study reports a superinfection rate of 13.9%. Presence of a superinfection significantly increases the likelihood of mortality within 28-days from admission. Characteristics that have a significant correlation to increased risk of superinfections include Black ethnicity, chronic kidney disease, ICU upon admission, and receipt of tocilizumab.

**Disclosures:**

**Kevin W. Garey, Pharm.D., M.S., FASHP**, **Summit Therapeutics** (Research Grant or Support)

